# Scholarship in Emergency Medicine: A Primer for Junior Academics: Part II: Promoting Your Career and Achieving Your Goals

**DOI:** 10.5811/westjem.2018.5.37539

**Published:** 2018-06-11

**Authors:** James Langabeer, Michael Gottlieb, Chadd K. Kraus, Shahram Lotfipour, Linda S. Murphy, Mark I. Langdorf

**Affiliations:** * University of Texas McGovern Medical School, Houston, Texas; † Rush University Medical Center, Chicago, Illinois; ‡ Geisinger Health System, Department of Emergency Medicine, Danville, Pennsylvania; § University of California Irvine Health School of Medicine, Department of Emergency, Irvine, California; # University of California Irvine, UCI Science Library Reference Department, Irvine, California

## Abstract

Scholarship is an important component of success for academic emergency physicians. Scholarship can take many forms, but all require careful planning. In this article, we provide expert consensus recommendations for improving junior faculty’s scholarship in emergency medicine (EM). Specific focus is given to promoting your research career, obtaining additional training opportunities, networking in EM, and other strategies for strategically directing a long-term career in academic medicine.

## INTRODUCTION

Think about the future, 30 years from now. What do you want to be doing in your academic career? Become a full-professor? Travel and speak internationally? Be responsible for the academic career path of many mentees? Own a body of literature or an innovation that has somehow changed medical practice or education? Be a clinical leader in your department, institution, or the government? Consult in academics or industry? Influence public policy? Train the next generation of physicians?

These goals are all realistic, but they require you to think strategically about your career and make periodic and detailed plans. In this second part of the primer series, we will offer tools to promote your academic career and achieve your goals. These include learning critical skills for peer review, understanding your academic track, focusing and promoting your research career through social media and other electronic means, and networking within emergency medicine (EM) organizations and societies.

## SCHOLARSHIP

To really succeed in academic EM, there must be a focus on scholarship. Scholarship can be defined in many ways, including excellence, higher learning, or achievement. Traditionally, scholarship was synonymous with research, but this definition is not adequate. The Carnegie Foundation for the Advancement of Teaching concluded in a landmark report that there needs to be a more comprehensive view of scholarship, and suggests that scholarship incorporates “a recognition that knowledge is acquired through research, synthesis, advanced practice, and teaching.”^1^ Scholarship as research focuses on discovery of new science that can guide future practice. Scholarship as applied practice focuses on quality improvement. Scholarship as synthesis (or integration) focuses on making connections and building different perspectives across multiple disciplines.^2^ Scholarship as teaching would identify strategies for improving instructional design, curriculum, and teaching processes. These four domains of scholarship are all potential paths to improving scholarship.^3^,^4^ Some might excel in practice or in teaching, while others in research or applied paths. Be sure to examine your own motivation behind your choices.^5^

All residents undertake a scholarly project during their residency training. The goals of the project are primarily to “instruct residents in the process of scientific inquiry,” but they often take multiple paths and shapes.^6^ Some are more focused on methodology and data, while others are more practical and applied. Improving your scholarship starts with the residency but continues throughout an academic career. In this article, we sought to incorporate expert consensus recommendations on improving scholarship in EM. We direct this primarily to residents, fellows, and junior faculty at universities and teaching hospitals who strive for long-term successful careers in academic EM.

## UNDERSTAND YOUR ACADEMIC TRACK

The form of scholarship you select is partially guided by the academic track you are in, as each track values research, teaching, service, and clinical endeavors differently.^7^ Previously mentioned, publication-related factors (e.g., impact factor, ranking of the journal within the specialty, authorship position of the faculty member) are important considerations for scholarly production for all academic tracks. Knowing the requirements set forth by the promotions and tenure committee helps you know by which criteria you will be judged. One direct resource for this would be your department’s vice chair or the school of medicine senior associate dean for faculty affairs.

Although academic tracks can vary among institutions, some factors remain common. The most common tracks to be considered are 1) clinical; 2) clinical scholar; and 3) tenure track. Another factor of importance is the institutional requirements, which may be independent of the specific academic track.

The clinical track encompasses the vast majority of faculty, and their record is evaluated by teaching, clinical effectiveness and efficiency. They can also be judged by the number of publications and their authorship position. First-author position is most important for assistant professors and final author or corresponding author for full professors.

The clinical scholars (educators) track criteria for promotion are not uniform among academic institutions, but they often have a requirement for focused original research as well. It is possible to achieve scholarly work in teaching of residents and students, as well as program evaluation of curriculum. Scholarship in this form requires different types of journals the faculty would be able to submit to and the corresponding difficulty in successfully being published. However, there are multiple types of scholarship beyond traditional publications. There are good articles that describe criteria and options for scholarship. Publications are not all the same, however. Original research articles are usually considered most scientifically significant and are at the top of the publication “hierarchy.” Systematic reviews and focused topic reviews fall just beneath this in the hierarchy. Case reports and images are typically less significant. Letters to the editor (although important in academic discussion) and book reviews might not be considered for promotion. Regardless of which publication type, we encourage junior faculty to get multiple articles in production early in their career.

The tenure track is the traditional research track with very little clinical responsibility and the majority of time dedicated to focused research. Criteria for promotion in this track are quite strict, requiring high-impact, first-author publications and significant grants with the National Institutes of Health or other government agencies. This track is only for faculty who strive to make a difference through a career in a very narrow field of research. The need for continued future research funding remains one of the greatest challenges for this very important academic track.

### Learning Critical Skills for Peer Review

Regardless of your specific academic pathway, scholarly activities are an essential component of the promotion and tenure process. While scholarship can involve a number of formats, often this involves conducting research and publishing. It should be noted that academic writing and communication is a distinct skill and markedly different from creative writing taught in high school and college.^8^ Academic writing focuses on the shortest direct prose that communicates the message. Journal editors strive to include as many papers as possible in the allotted pages of an issue. Therefore, brief, concise, to-the-point articles are preferred. Note that Watson and Crick’s hallmark paper on the structure of DNA was only 1.5 pages in length, proving that a paper need not be long to be impactful.^9^

How do you develop this skill? There are only two ways: practice writing academic papers and read scientific papers written by other authors. Volunteering your time as a peer reviewer offers a vital service to academic medicine and society, while improving your own writing. Most developing journals welcome junior reviewers after they have shown a modest number of publications in a specific specialty. Another way to become a reviewer is to be recommended by a senior faculty member who is known to the editor-in-chief or senior editors on the journal. Peer review can be a difficult and time-consuming process—a well-done review can take two or more hours—but your topic expertise and writing skills will grow through experience.^10^ There is an excellent tutorial from the *Annals of Emergency Medicine* on the peer-review process for those interested in learning further.^11^

Peer review is an extension of the training you received in journal club during residency. Your task is to judge whether the authors properly framed the research question and used the appropriate study design; additionally, to the extent of your knowledge, your task is to critique the statistical methods employed, identify confounding factors and limitations, and comment on the appropriateness of the conclusions based upon the study’s data. Most importantly, you should analyze the presentation of the information such that a clinician can understand the paper and modify practice, if indicated. If you didn’t understand the author’s logic or argument, then it is unlikely others will.^12^

Although there are certainly expert reviewers with formal training in methodology and statistics, this is not required for the average reviewer. Journals typically have methodology experts on staff to analyze and critique these components. The editor is most interested in whether the paper is clear, concise, well-organized, appropriately novel, and that the authors performed an appropriate literature review. Any junior scientist can certainly fill that role. Think about what you understood from their argument, and what could have been presented more clearly. When you read another’s writing, you will quickly discover pitfalls and areas of confusion with how the information is presented. However, just as this is valuable to share with the author, it is also useful for you when you write your next paper, as you will avoid the same mistakes.

When you apply for promotion in the academic hierarchy, this service as peer reviewer is valued as a demonstration of commitment to your specialty, community, and science in general. Keep track of the journals for which you have reviewed, and the number of reviews. Many journals provide recognition for top quality and quantity, and publish such names on their websites and within their pages.

### Promoting Your Scholarship

One of the most important milestones for academic emergency physicians is the publication of your first article. It’s a time to celebrate. However, it is important to note that this is not the end. Once you get the manuscript published, it’s important to make it available for others to read. While anonymity has a role in some things, academic promotions are based on visibility and impact. This requires you to think about how to best disseminate your findings and “promote” the scholarship to gain visibility and prestige. Consider how best to translate your findings, so that other clinicians and the community in general can benefit. While visibility is essential to transferring research into practice, it is equally important for your academic promotion and tenure. This is becoming increasingly challenging given the huge amount of scientific information being published today in EM.^13^ Your scholarship (whether it’s research, teaching, application, or innovation) needs to be seen and read.

Here we discuss five strategies to promote your scholarship. These include: 1) presentations; 2) collaborations and citations; 3) social media and blogs; 4) open access repositories; and 5) institutional and personal platforms.

The traditional route to achieving visibility after publication is to present your research at society meetings and conferences in abstract form. Presenting at regional and national conferences is a great way to share your findings. Presenting will also help you meet others in your research area, which can lead to future collaborations and research ideas. Building practical collaborations and research partnerships are additionally useful for designing future projects and obtaining funding. You should note that most research is collaborative in nature. The largest clinical research studies—such as SIREN (Strategies to Innovate EmeRgENcy Care Clinical Trials Network) or PECARN (Pediatric Emergency Care Applied Research Network)—are large collaborations between researchers at multiple institutions. Developing research projects on your own is very time-consuming; networks help to build sufficient critical mass and increase the number of patients to demonstrate external validity. Collaboration is vital to getting involved with projects, receiving funding, and publishing significant results.

In addition, through collaboration multiple individuals share the task of promoting your works. This helps broaden the network and provide greater visibility. In the process, be sure to cite yourself and others in the field. Citations themselves can also serve as promotion tools. When you cite others, it shows that you understand the current research. Additionally, when you share your findings with researchers you cited, others get to know you and your research, further increasing your available collaboration network. As editors and senior researchers (those who make decisions) become familiar with your research, they will likely cite you as well. Most journal editors enjoy publishing the work of established names in research.

Some journals offer authors the option of recording an audio or video summary to be posted on the journal website. If offered, avail yourself of this opportunity, as it can further increase your academic profile and article visibility. Another strategy to engage with researchers is to join academic society committees or interest groups, and meet others doing research in your field. Both have websites and email listservs, which can share collaboration ideas and grant opportunities. You may also want to attend research conferences outside of EM, to further interface with researchers in your area of interest. It would also be beneficial to consider attending conferences in subspecialties, such as cardiology, education, emergency medical services, geriatrics, neuroscience, public health, pediatrics, toxicology, and trauma to name a few.

The use of online social media is becoming increasingly common within EM, with platforms such as Twitter, Facebook, and LinkedIn allowing researchers to engage larger audiences through simplified messages. Twitter especially has emerged as a method of distributing research findings. A recent study published in the *Western Journal of Emergency Medicine: Integrating Emergency Care with Population Health* (*West*JEM*)*, examined the most influential and connected emergency physicians in the country, discovering that certain physicians who are highly active on social media serve as major influencers in medical education.^14^ Another study shows strong correlation between tweets and citations, especially in the first few days after publication.^15^ Frequently-tweeted papers are many times more likely to be highly cited.^16^ As an example of its power, Twitter enabled the International Conference on Emergency Medicine (ICEM) in 2012 to become the most tweeted EM conference on record.^17^

Additional resources include LinkedIn, Facebook, and Doximity, which enable others to find you and connect for future projects. Social media portfolios have been shown to be instrumental in the promotion and tenure process.^18^ Alternatively, the use of free open access medical education (FOAM) resources, such as blogs and podcasts, can be useful in further defining one’s niche.^19^ Many EM faculty maintain blogs and share their findings with others from the field.

Self-archiving through open access repositories, such as ResearchGate, Academia.edu, Mendeley, Kudos and other sites, provides a wider circulation of published work. Institutional repositories and websites also provide an online presence in perpetuity. This is why it is important to actively update and maintain your faculty websites, personal websites, and biographies so others can find your work through search engines. The use of Google Scholar as a research tool has expanded greatly so maintaining updated biographies and research interests helps ensure that searches find your articles. We encourage all junior faculty to create a Google Scholar profile. You have the option to either make your profile public or keep it private. It obviously provides much greater visibility to make it public.

Gaining visibility for your research career requires a strategy. It is important to plan in advance of your publication and continue to highlight your research. The above strategies can help to increase your success.

### Pursue Additional Training

Achieving additional levels of training can help improve your scholarship and your career. You can do this in a number of ways. Some might consider the American College of Emergency Physicians (ACEP) teaching fellowship, which is designed to improve skills of instruction. The Council of Emergency Medicine Residency Directors offers a medical education research certificate program (MERC), which also focuses on faculty and educational development. Some physicians might choose to pursue additional graduate degrees or certificates in other areas to extend their niche. Fellowships in anything from clinical research to education or policy might be interesting to consider. Advanced training, whether in health services research, medical informatics, public health, business administration, or even secondary clinical specialties, is a factor to consider as a means to improve scholarly capacity. Base the decision on your interests, skills, and long-term vision of your own career.

### Collaboration and Networking

For those early in their academic career, it is critical to appreciate the value of networking with colleagues in EM societies, organizations, and conferences. Networking is simply connecting with others to build relationships, exchange professional experiences and ideas, collaborate around topics of mutual interest, and develop contacts that go beyond your traditional hospital borders. Networking has been shown to be valuable for making new-hire decisions, securing research funds, and ensuring overall career success.^20^ When you visit one of the major academic assemblies, be sure to set up meetings in advance or arrange for coffee to introduce yourself to others from your specialty. SAEM offers junior faculty development workshops, and ACEP has a wide variety of events, receptions, and workshops in which you can participate. The exhibit halls are also a great way to introduce you to new concepts. When you get back from the event, be sure to follow up and stay in touch with the people you have just met.^21^

The time you spend meeting your peers with similar interests and research is invaluable to developing and promoting your career. Nearly every professional society has networking events where you can connect with others with whom you might form research or clinical relationships. Seek out opportunities for leadership roles in your local and state EM organizations. Seek out senior faculty who have conducted interesting research or who work in an area you would like to learn more about. Since most conferences publish the list of attendees well in advance, you can usually find out who will be attending and approach them by email or phone well in advance. When you go to a research presentation, stay afterward and ask questions of the presenters. Network and exchange business cards with others who do the same.

Lastly, you should form a mentor relationship with someone senior in your field, especially if you are considering a focused research or clinical subspecialty in EM. Mentors are very important for the academic emergency physician, offering access to different professional experiences and existing networks. There are many choices you will make along the way (e.g., which journal to submit to, which research to pursue, which job offer to take). Before making these choices you could benefit from the expertise of more-senior colleagues. Additionally, securing a mentor can be valuable when applying for grants and collaborating without outside departments and institutions. When seeking out mentorship, ensure that your goals align and that the mentorship relationship is a good fit. For further information, junior faculty are encouraged to review the excellent summary by Straus and colleagues.^22^

## CONCLUSION

Scholarship in emergency medicine should be broadly focused. While research is the most traditional path, it is equally as important to consider excelling in the scholarship of application, integration, or teaching. It is important that junior faculty conduct periodic planning and develop both short- and long-term plans outlining directions and goals for their career. If your goal is to build a career in academic EM, be sure to focus on promoting and bringing visibility to yourself and your scholarship. Remember there are differences by academic track, which might influence the type of scholarship you pursue. In all areas, be sure to include peer reviews, mentorship, networking, and social media to expand your visibility and knowledge.

## References

[1] Boyer E (1990). Scholarship Reconsidered: Priorities of the Professoriate.

[2] Hofmeyer A, Newton M, Scott C (2007). Valuing the scholarship of integration and the scholarship of application in the academy for health sciences scholars: recommended methods. Health Res Policy Syst.

[3] Glassick CE (2000). Boyer’s expanded definitions of scholarship, the standards for assessing scholarship, and the elusiveness of the scholarship of teaching. Acad Med.

[4] Chan TM, Gottlieb M, Fant AL (2016). Academic Primer Series: Five Key Papers Fostering Educational Scholarship in Junior Academic Faculty. West J Emerg Med.

[5] Borges NJ, Navarro AM, Grover A (2010). How, when, and why do physicians choose careers in academic medicine? A literature review. Acad Med.

[6] Summers RL, Fish S, Blanda M (1999). Assessment of the “scholarly project” requirement for emergency medicine residents: report of the SAEM Research Directors’ workshop. SAEM Research Directors’ Interest Group. Acad Emerg Med.

[7] Regan L, Stahmer S, Nyce A (2010). Scholarly tracks in emergency medicine. Acad Emerg Med.

[8] Langdorf MI, Hayden SR (2009). Turning your abstract into a paper:academic writing made simpler. West J Emerg Med.

[9] Watson JD, Crick FH (1953). Molecular structure of nucleic acids; a structure for deoxyribose nucleic acid. Nature.

[10] Garg N, Gottlieb M (2018). Resident research tips: surviving and thriving within the peer review process. Ann Emerg Med.

[11] Callaham MSD, Cooper RJ (2017). An Instructional Guide for Peer Reviewers of Biomedical Manuscripts.

[12] Young JM, Solomon MJ (2009). How to critically appraise an article. Nat Clin Pract Gastroenterol Hepatol.

[13] Trueger NS, Thoma B, Hsu CH (2015). The altmetric Sscore: a new measure for article-level dissemination and Impact. Ann Emerg Med.

[14] Riddell J, Brown A, Kovic I (2017). Who are the most influential emergency physicians on Twitter?. West J Emerg Med.

[15] Eysenbach G (2011). Can tweets predict citations? Metrics of social impact based on Twitter and correlation with traditional metrics of scientific impact. J Med Internet Res.

[16] Fitzgerald RT, Radmanesh A (2015). Social media and research visibility. AJNR Am J Neuroradiol.

[17] Neill A, Cronin JJ, Brannigan D (2014). The impact of social media on a major international emergency medicine conference. Emerg Med J.

[18] Cabrera D, Vartabedian BS, Spinner RJ (2017). More than likes and tweets: creating social media portfolios for academic promotion and tenure. J Grad Med Educ.

[19] Stuntz R, Clontz R (2016). An evaluation of emergency medicine core content covered by Free Open Access Medical Education Resources. Ann Emerg Med.

[20] Gottlieb M, Sheehy M, Chan T (2016). Number needed to meet: ten strategies for Improving resident networking opportunities. Ann Emerg Med.

[21] Gottlieb M, Fant A, King A (2017). One click away: digital mentorship in the modern era. Cureus.

[22] Straus SE, Johnson MO, Marquez C (2013). Characteristics of successful and failed mentoring relationships: a qualitative study across two academic health centers. Acad Med.

